# The effect of laboratory diet and feeding on growth parameters in juvenile zebrafish

**DOI:** 10.1038/s41684-024-01456-6

**Published:** 2024-10-23

**Authors:** Courtney Hillman, Austin H. Cooper, Pooja Ram, Matthew O. Parker

**Affiliations:** Surrey Sleep Research Centre, University of Surrey, Guildford, UK

**Keywords:** Animal physiology, Scientific community

## Abstract

Despite being one of the most used laboratory species in biomedical, behavioral and physiological research, the nutritional requirements of zebrafish (*Danio rerio*) are poorly understood, and no standardized laboratory diet exists. Diet and feeding regimen can substantially impact the welfare of the fish and, in turn, experimental reproducibility. Consequently, the establishment of a standardized diet and feeding protocol for laboratory zebrafish is imperative to enhance animal welfare, guarantee research reproducibility and advance the economic and environmental sustainability of laboratory dietary practices. Here the aim of this systematic review is to provide an overview of the parameters that need to be standardized in future nutritional studies to facilitate future meta-analyses for confirmation of an optimal juvenile diet for growth. A comprehensive search was conducted in PubMed and Scopus to identify relevant studies published up to August 2023, and the studies were selected on the basis of the predefined inclusion/exclusion criteria. The databases yielded a total of 1,065 articles, of which 14 were included in this review. We conducted data extraction and risk-of-bias analysis in the included studies. Statistical comparisons for specific growth rate, weight gain (%) and length gain (%) parameters were performed to determine the optimal feed for enhanced juvenile growth. We identified significant heterogeneity and caveats to our findings owing to a lack of standardization of experimental conditions in nutritional studies. Our findings highlight an urgent need for research on zebrafish nutrition. Therefore, the standardized parameters we have reported here represent a critical starting point for studies.

## Abstract (web summary)

This systematic review identifies substantial heterogeneity in the experimental conditions in zebrafish nutrition studies, highlighting the urgent need for further research and the importance of standardized parameters for future zebrafish studies.

## Main

Zebrafish (*Danio rerio*) are one of the most common research organisms in the fields of behavior, genetics, physiology and biomedical science, with high genetic homology to mammals, low husbandry costs, rapid breeding and an ease of genetic manipulation^1–4^. However, despite their increasing use in research, there is a lack of a standardized feeding regimen and knowledge on nutritional requirements for juvenile growth and development^5–8^. The lack of consistent diet used between research groups can substantially affect fish welfare and increase experimental inconsistencies^5,8,9^. It is therefore essential that a standardized diet be established that promotes the welfare of the zebrafish with the added benefit of improving experimental robustness and facilitating experimental global collaboration.

A lack of understanding of the requirements of optimal zebrafish nutrition has limited the ability to determine a standardized laboratory diet^10^. Currently, the dietary requirements of laboratory zebrafish are related to published information on other species of fish that demonstrate similar feeding habits or live in comparable habitats to wild zebrafish^5,11–13^. *The Zebrafish Book* by Westerfield^14^ describes the feeding requirements of zebrafish to be a variety of commercial and/or trout pellets with enough feed supplied twice a day for each fish to feed and all food to be eaten within 5 min (ref. ^14^). Although generic nutritional components of the zebrafish diet are agreed upon (proteins, carbohydrates, lipids, vitamins and minerals), there is currently no consensus on the optimal overall diet composition. Therefore, it is essential to determine a standardized laboratory diet through establishing the nutritional requirements of zebrafish to ensure reproducible research and to promote welfare.

In this systematic review, we aimed to establish the optimal feed for three different parameters of juvenile growth (specific growth rate (SGR), length gain and weight gain) in zebrafish. To determine this, we performed a systematic review of the available scientific literature studying the effects of diet on growth in juvenile zebrafish. We provide an overview of the current literature relating to zebrafish growth responses with different feeds by providing a qualitative description of the published studies as well as evaluating the impact of bias arising from methodological conduct, reporting quality and selective publication. In summary, our aim initially was to provide a comprehensive and data-driven approach to investigate the following research question: what feed type is optimal for juvenile zebrafish growth? In the process of answering this question, however, it was clear that the publications relating to juvenile zebrafish growth lacked sufficient experimental standardization for effective analyses to take place. Therefore, the revised aim of this systematic review is to provide an overview of the parameters that need to be standardized in future nutritional studies to facilitate future meta-analyses for confirmation of optimal juvenile diet for growth.

## Results

### Search results

**Fig. 1:**
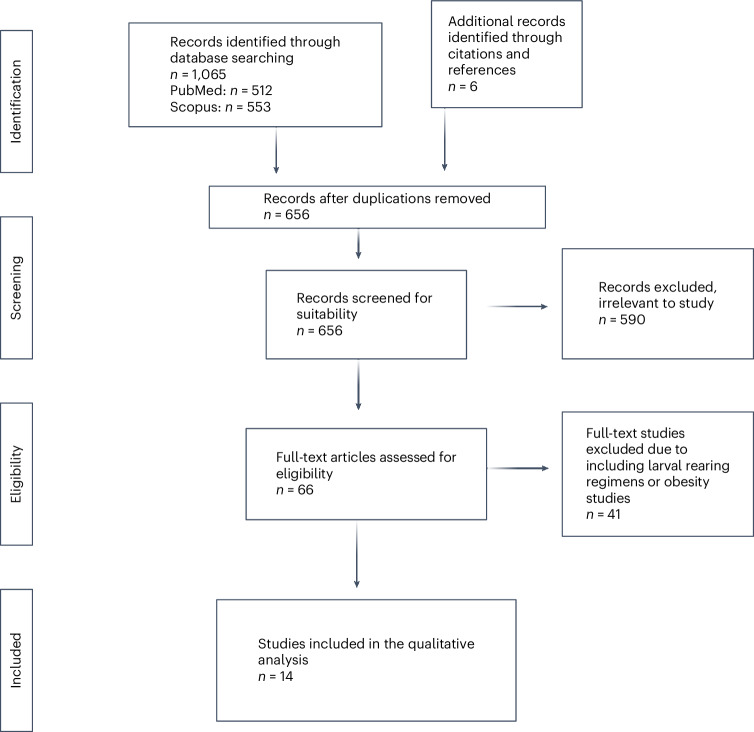
Flowchart diagram of the collection of studies and selection process. The Preferred Reporting Items for Systematic Reviews and Meta-Analyses (PRISMA) flow diagram for the systematic review detailing the database searches, the number of abstracts and full texts screened, the number of reports retrieved and the number of studies included in the review.

Database searches resulted in an initial 1,065 documents (*n* = 512 from PubMed and *n* = 553 from Scopus) through systematic searches (Fig. 1). Duplicates (*n* = 656) were removed, and studies with unrelated research questions, different research models and other study types were excluded (*n* = 590). After these exclusions, 66 articles remained, which were subjected to a full-text reading. Any studies that exclusively used zebrafish <28 days post-fertilization (dpf), incorporated supplements into the diet for translational purposes, or over- and/or underfed the fish for obesity studies were excluded (*n* = 41). After selection by title, abstract and full text, 25 original research articles with adult zebrafish ≥28 dpf testing the effects of feed on growth were obtained. Fourteen of these studies were included in the present systematic review, and the main characteristics are found in Table 1 and additional extracted data in Supplementary Table 1. Nine studies were not included after having been initially deemed suitable, four of which were excluded because they produced substantial publication bias (*n* = 1) or were the wrong age for the parameters for which they presented data (*n* = 3)^9,15–17^. Four of the studies that were excluded assessed reproduction; however, this number was below our threshold for inclusion for statistical comparison, and therefore, the studies were not included any further in the analysis^18–21^. Briefly, these excluded reproduction studies found a statistically significant difference in egg production and fry survival with different experimental diets, including feeding supplements^18–21^. Therefore, further studies analyzing the effect of zebrafish feed on reproduction are required for sufficient comparison and analysis.

**Table 1:** Qualitative description of studies reporting growth-related effects of experimental diets on juvenile zebrafish

Author	Age at the start of study to end of study (dpf (or months if specified))	Sex (male:female)	Strain	Initial weight (mg ± s.e.m.)	Initial length (mm ± s.e.m.)	Number of fish per experimental replicate	Tank density per liter	Diet(s) pre-experiment	Experimental diet(s)	Feeding regimen	Housing conditions
Barca et al.^22^	60–102	N/A	AB	43.6 ± 22.96	N/A	80	5.7	N/A	D1: 50% FM; D2: 17% insect meal + 33% FM; D3: 33% insect meal + 17% FM; D4: 50% insect meal	Ad libitum according to ‘five-minute rule’ for 42 days. Feed was 400–600 μm PS	Water temperature: 28 ± 0.5 °C; pH: 7.2–7.8; electrical conductivity: 600 and 800 µS/cm; dissolved oxygen: >5 mg/l; ammonia: <1 mg/l; nitrites: <0.25 mg/l; nitrates: <50 mg/l; photoperiod: 12:12 L:D; water flow: 2 l/h
Fredrickson et al.^38^	29–91 (growth at 72)	1:1	EK	N/A	15.31 ± 0.5	572	10.66	5–9 dpf: type-L saltwater rotifers; 10–28 dpf: brine shrimp 2/day + larval AP100 1/day + Hatchfry Encapsulon 3 1/day	D1: Ocean Star International flake food + freeze-dried krill 3:1; D2: GM300 + brine shrimp	D1: 2/day + 2/day brine shrimp (0.2 g/10 fish); D2: 1/day (10 mg/10 fish) + brine shrimp (0.2 g/10 fish)	Water temperature: 26.7 °C; pH: 7.0; electrical conductivity: 1,000 µS/cm; dissolved oxygen: 7.30 mg/l; photoperiod: 14:10 L:D
Fronte et al.^34^	60–109	N/A	AB	85.3 ± 25.58	N/A	60	N/A	4/day with commercial feed + brine shrimp until satiation following the ‘five-minute rule’	D1: 20% FM; D2: 5% HIM + 15% FM; D3: 10% HIM + 10% FM; D4: 20% HIM	4/day to satiation for 49 days	Water temperature: 28 ± 0.5 °C; electrical conductivity: 600–800 µS/cm; dissolved oxygen: >7 mg/l; ammonia: 1 mg/l; nitrite: 0.25 mg/l; nitrate: 50 mg/l; photoperiod: 12:12 L:D
Lanes et al.^35^	30–90	N/A	Other^A^	5.0 ± 0.5	9.2 ± 0.3	100	6.25	N/A	D1: 50% FM; D2: defatted V instar larvae meal; D3: defatted prepupae meal	3/day ad libitum for 60 days; days 1–5: <150 μm PS; days 16–35: 150–250 μm PS; days 36+: 300–400 μm	Water temperature: 27 ± 0.3 °C; pH: 8.1 ± 0.1; dissolved oxygen: 6.4 ± 0.12 mg/l; photoperiod: 12:12 L:D
Samuel et al.^27^	Uniform-sized adult	N/A	Other^B^	N/A	N/A	10–12	N/A	N/A	D1: soybean-based diet; D2: FM; D3: probiotic-based diet; D4: combination; D5: crude protein	1/day 4% BW for 30 days	N/A
Vural et al.^31^	2 months	1:1	Other^B^	350 ± 150	N/A	50	N/A	N/A	D1: casein; D2: 0.10% RJ; D3: 0.40% RJ; D4: 1.60% RJ; D5: 6.40% RJ	4/day at 5% BW for 56 days. Feed size between 1 and 2 mm diameter	Water temperature: 26 ± 1 °C; pH: 7.3 ± 0.3; dissolved oxygen: 9 ± 0.5 mg/l; photoperiod: 14:10 L:D
Carneiro et al.^23^	30–90	1:1	Other^C^	163 ± 2.9	N/A	75	5	2/day for 2 weeks with flocculated commercial feed	D1: 50% FM; D2: 40% FM, 10% CS; D3: 30% FM, 20% CS; D4: 20% FM, 30% CS; D5: 10% FM, 40% CS; D6: 50% CS	3/day until satiation for 60 days. Feeds were sieved through 0.3 mm sieve and mixed using 4–6 mm extruders	Water temperature: 27.8 ± 0.6 °C; pH: 7.4 ± 0.3; dissolved oxygen: 7.9 ± 0.5 mg/l; ammonia: 0.09 ± 0.05 mg/l; photoperiod: 14:10 L:D
da Silva et al.^24^	50–105	1:0	N/A	290 ± 40	30.67 ± 0.71	60	1.2	N/A	D1: SPI; D2: 3% FO + 0.5% CLEO; D3: 3% FO + 1% CLEO; D4: 6% FO + 0.5% CLEO; D5: 6% FO + 1% CLEO; D6: 9% FO + 0.5% CLEO; D7: 9% FO + 1% CLEO	4/day until satiation for 55 days. Feed size was 1 mm in diameter	Water temperature: 26.23 ± 0.54 °C; pH: 7.3 ± 0.01; dissolved oxygen: 6.6 ± 0.32 mg/l
Dhanasiri et al.^30^	4 months	1:1	AB	214	N/A	32	4.57	Live feed first + commercial feed	D1: 79.4% FM; D2: pea protein concentrate + FM; D3: WG + FM; D4: SPC + FM	2/day 2.5% (w/w) of BW for 46 days	Water temperature: 28 ± 0.5 °C; pH 7.5; electrical conductivity: 1,500 μS/cm; photoperiod: 12:12 L:D
Sevgİlİ et al.^29^	35	N/A	Other^D^	88.61 ± 0.82	N/A	90	3	Acclimated diet of commercial rainbow trout	D1: 19.87 FM; D2: 26.49 FM; D3: 30.90 FM; D4: 37.51 FM; D5: 41.92 FM; D5: 48.54 FM; D6: 52.95 FM; D7: 59.56 FM	Ad libitum 2/day for 4–6 weeks. Feed was extruded into 2 mm PS	Water temperature: 24.87 ± 0.49 °C; pH: 8.52 ± 0.06; dissolved oxygen: 7.65 ± 0.06 mg/l; ammonia: <0.02 mg/l; nitrites: 0.013 ± 0.003 mg/l; photoperiod: 13–14:11–10 L:D
Fernandes et al.^26^	54	N/A	Other^B^	53.6	17.8	20	18	Brine shrimp up to 30 dpf + TetraMin flake feed 30–54 dpf	D1: 15% FM; D2: 20% FM; D3: 25% FM; D4: 30% FM; D5: 35% FM; D6: 40% FM; D7: 45% FM; D8: 50% FM; D9: 55% FM; D10: 60% FM	Fed to satiation 2/day 6 days/week for 8 weeks. Feed was between 400 and 600 μm PS or 600 and 1,000 μm PS	Water temperature: 28 °C ± 1 °C, pH: 8.2, photoperiod: 14:10 L:D
Karga & Mandal^36^	2 months	1:1	N/A	249 ± 4	N/A	60	2.5	N/A	D1: zooplankton; D2: 35% FM; D3: 40% FM; D4: 45% FM	4% BW for 90 days, then 3% BW for 210 days. Feed was sieved through 0.1–0.3 mm for small PS	Water temperature: 27–28 °C; pH: 6.8–7.4, dissolved oxygen: 6–8 mg/l; ammonia: 0.01–0.6 mg/l; photoperiod: 12:12 L:D
Smith et al.^28^	28	N/A	AB	N/A	15.7 ± 0.21	50	5.5	5–28 dpf: *Brachionus plicatilis* rotifer ad libitum enriched with *Nannochloropsis* 3/day	D1: FPH; D2: casein; D3: SPI; D4: WG; D5: mix (FPH, casein, SPI, WG)	3/day at 5% BW for 16 weeks. All feed provided as a power with PS 250–500 μm	Water temperature: 28 °C; pH: 7.4; electrical conductivity: 1,500 μS/cm
Lawrence et al.^25^	30–191	1:1	AB	70 ± 10	19.2 ± 0.6	140	11	5–9 dpf Tyle L saltwater rotifer (*Brachionus plicatilis*) ad libitum 10–30 dpf brine shrimp 3/day to satiation	D1: Art.; D2: GM300 1; D3: GM300 2; D4: GM300 3; D5: GM300 4	D1: to satiation 3/day; D2: 5% BW 1/day; D3: 1.65% BW 3/day; D4: 1% BW 5/day; D5: 5% BW 1/2 days; for 161 days	Water temperature: 26.69 ± 0.10 °C; pH: 7.26 ± 0.02; electrical conductivity: 1,291.69 μS; dissolved oxygen: 7.9 ± 0.16 mg/l; nitrites: 0.02 ± 0.001 mg/l; nitrates: 2.79 ± 0.22 mg/l

BW, body weight; CS, *Chlorella spirulina*; D(…), diet(number); FPH, fish protein hydrosylate; HIM, *Hermetia illucens* meal; L:D, light:dark; N/A, not applicable; Other^A^, external supplier; Other^B^, pet-store zebrafish; Other^C^, short-fin zebrafish; Other^D^, defined by authors as pink-type; PS, particle size; RJ, Royal Jelly; SPC, soy protein concentrate; SPI, soy protein isolate; WG, wheat gluten.

### Study characteristics

**Fig. 2:**
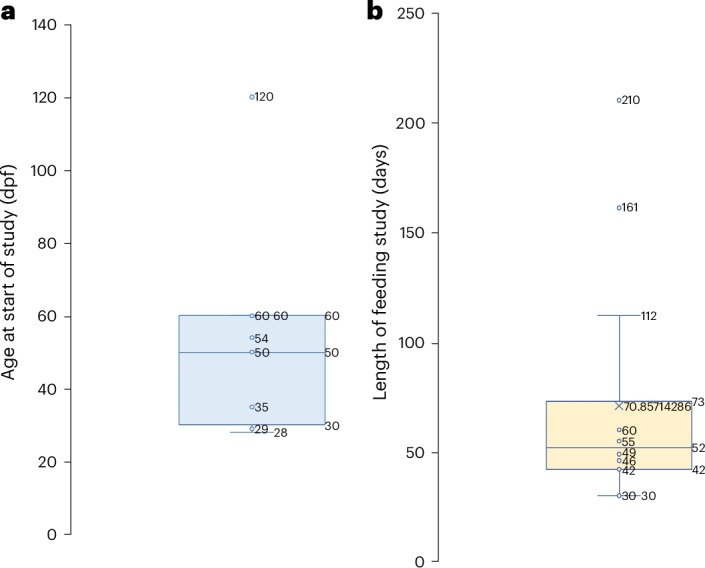
Comparison of the age of the zebrafish at the start of the feeding trial and length of feeding trial represented using box-and-whisker plots. **a**,**b**, Box-and-whisker plots comparing the age at the start of the feeding trial (**a**) and the length of feeding trial (**b**).

The studies included in this systematic review were published between 2012 and 2023. These studies were carried out in the United States (*n* = 3), Italy (*n* = 2), Turkey (*n* = 2), India (*n* = 3), Brazil (*n* = 2), France (*n* = 1) and Portugal (*n* = 1). The ages of fish ranged from 28 dpf to 4 months at the start of the feeding trial, and trial length ranged from 30 to 210 days (Fig. 2). A breakdown of the diets is presented in Table 2.

**Table 2:** The different feeds, supplements and experimental diets used in the articles including the feeding category and the number of groups using this feed (*n*)

Feed	Category	*n*
GM300	1	4
	6	1
Ocean Star International Flake Feed (Hung Ling Int.) + krill	6	1
FM	3	27
Insect-based diets	4	8
Pea protein	2	1
Soy protein	2	4
Wheat gluten	2	2
Artemia/krill/zooplankton	3	2
Fish protein hydrolysate	3	1
Casein	3	2
Mix	6	2
Probiotics	5	1
Royal Jelly	5	4
*Chlorella sp*.	5	5
*Streptomyces platensis*	5	1
FO and clove leaf	5	6

Overview of the categories of each feed included in the analysis as well as the number of groups using the feed. Category 1, commercial feed; category 2, plant-based protein; category 3, animal-based protein; category 4, insect-based protein; category 5, supplements and additives; category 6, mix/combination feeds.

**Fig. 3:**
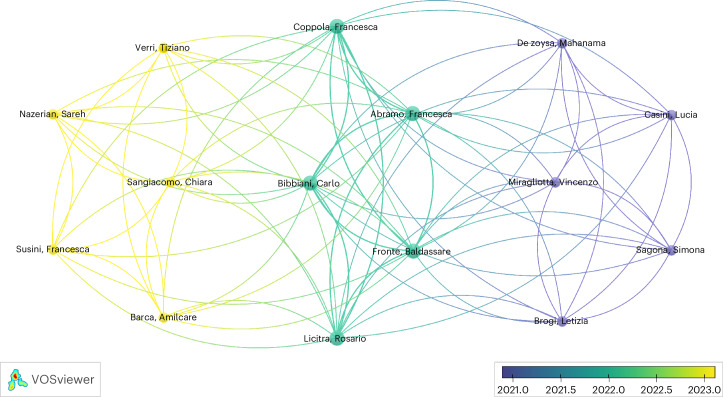
Co-authorship network analysis of researchers that authored studies assessing the effect of diet on growth. Authors are color-coded from violet (older studies) to yellow (most recent studies) indicating the average publication year of the studies published by each researcher. The size of the circles represents the number of studies published by each author. The distance between the two circles indicates the correlations between researchers.

The studies reported significant effects on juvenile growth with differing zebrafish feeds with no effect on overall survival observed^22–25^. A common finding among authors was a positive correlation between protein content, probiotics, *Chlorella spirulina* (*Chlorella sp*.) concentration or insect-based diets and body weight^22,23,26,27^. Similar findings were reported for fish length. However, feed intake was reported to decrease with increasing protein content, while a positive relationship was observed between body weight and lean body mass with the diets^26,28,29^. In addition to growth and survival parameters, Dhanasiri et al.^30^ reported moderate transcriptome changes in fast-muscle samples in zebrafish fed plant-based diets compared with animal-based diets^30^. Vural et al.^31^ also highlighted the upregulation of growth hormone genes with Royal Jelly supplementation compared with an animal-based diet^31^. A description of the studies included in the review can be found in Table 1. More detailed information at the study level for the variables extracted is available in Supplementary Table 1. The co-authorship network analysis can be found in Fig. 3, showing collaboration between researchers or research groups relating to feeding effects on zebrafish growth^32^. Limited collaboration was identified; see the interactive version of this co-authorship analysis for further information (www.vosviewer.com)^33^.

### Risk of bias

**Fig. 4:**
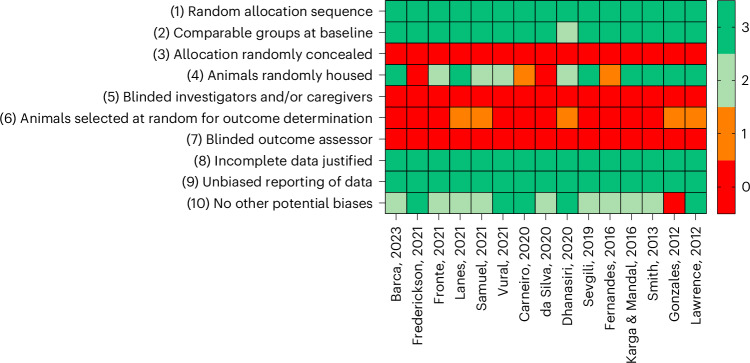
Risk-of-bias assessment of the included studies. The risk-of-bias assessment was performed by one independent investigator on the basis of the Systematic Review Centre for Laboratory Animal Experimentation (SYRCLE) risk-of-bias assessment tool. Red indicates high bias, orange is moderate, light green is slight and green indicates low bias. The numbers indicate how many researchers agree (3, low bias; 0, high bias).

The overall risk of bias for the items evaluating the methodological quality of included studies was considered low, except for items 3–7 and 10 (Fig. 4). Consequently, the overall publication risk of bias presented here is considered unclear. For items 3, 5 and 7, 100% of the studies provided insufficient data to rule out biases arising from the allocation of the animals to experimental groups, or biases resulting from investigators. Items 4 and 6 had moderate bias, with 26.6% and 86.6% of the studies, respectively, providing insufficient information to rule out the potential of biases impacting results owing to random housing of experimental animals and random outcome selection. The study by Gonzales^16^ was found to have a high risk of bias for item 10 due to 50% of the experimental diets incorporating feeds that are no longer commercially available^16^. Therefore, this study was removed from further analysis to ensure the results are of commercial relevance. In addition, Samuel et al.^27^ provided insufficient information regarding the age of fish, with age defined as ‘uniform-sized adults’ as well as a lack of husbandry condition reporting^27^. However, this study was included in the final analysis. The eight other additional potential risks were for Barca et al.^22^, Fronte et al.^34^, Lanes et al.^35^, Sevgili et al.^29^, Fernandes et al.^26^ and Smith et al.^28^, who did not include a sex split; and for da Silva et al.^24^ and Karga and Mandal^36^, who did not include the strain of fish^22,24,26,28,29,34–36^. These studies pose a potential risk of bias, which must be taken into consideration during analysis and interpretation of results. Out of the 450 scores for risk of bias, there were 21 (4.7%) disagreements between the three independent investigators. Of the 21 disagreements, 1 (4.8%) was for item 2, 6 (28.6%) were for item 4, 5 (23.8%) were for item 6 and 9 (42.8%) were for item 10.

### Growth parameters

**Fig. 5:**
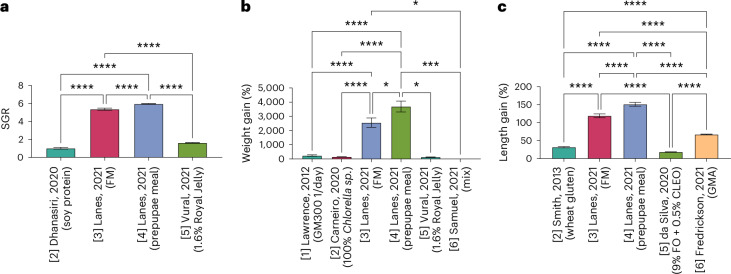
The overall growth analysis for the three growth parameters. **a**–**c**, The overall growth performance for each feeding category analyzed using a one-way ANOVA for SGR (**a**), weight gain (%) (**b**) and length gain (%) (**c**). GMA, GM300 and *Artemia nauplii*; 1/day, once per day; [number], category. Data are presented as mean ± s.e.m. Significance values are set at ****P < 0.0001, ***P < 0.001, **P < 0.01, *P < 0.05.

Zebrafish growth and development is greatly impacted by feeding regimen and protein levels; however, no standardized laboratory diet exists so far^8,37,38^. Therefore, here we aimed to determine the optimal feed type for juvenile larval growth by performing subcategory analyses within each feeding category (Methods and Table 2) followed by an overall statistical analysis to determine the optimal category (Fig. 5). Optimal growth was defined here as the largest SGR, greatest percentage weight gain and percentage length gain. Lanes et al.^35^ reported significantly greater SGR, percentage weight gain and percentage length gain than all other analyzed papers, which may have influenced the findings notably^35^.

### SGR

Subgroup analysis for category 3 and category 5 with regard to SGR revealed 50% fishmeal (FM) and 6.4% Royal Jelly to be optimal within each category, respectively^31^^,35^ (Supplementary Fig. 1). These feeds were then analyzed against soy protein isolate (category 2) and defatted prepupae meal (category 4) in the overall analysis^30,35^. This analysis revealed a significant effect of diet on zebrafish SGR (one-way analysis of variance (ANOVA): *F*_(3, 233)_ = 311.1, *P* < 0.0001), with the optimal diet identified as category 4, defatted prepupae meal^35^ (Fig. 5a).

### Percentage weight gain

Subgroup analysis for categories 2–5 with regard to percentage weight gain revealed 100% *Chlorella sp*. (category 2), 50% FM (category 3), 50% defatted prepupae meal (category 4) and 6.4% Royal Jelly (category 5) to be optimal within each respective category. The final analysis also included once per day Gemma Micro 300 (GM300) (category 1) and a protein mix (category 6)^23^^,25,27,31,35^ (Supplementary Fig. 2). The overall analysis revealed a significant effect of diet on weight gain in juvenile laboratory zebrafish (one-way ANOVA: *F*_(5, 424)_ = 32.09, *P* < 0.0001) (Fig. 5b). Further analysis with Tukey’s multiple comparison test revealed an insect-based diet (defatted prepupae meal) to be optimal for percentage weight gain compared with all other feeding categories, closely followed by an animal-based diet with 50% FM^35^. No significant difference was seen between the other feeding categories^23,25,27,31^.

### Percentage length gain

Subgroup analysis revealed 50% FM (category 3) and GM300 combined with brine shrimp (category 6) to be optimal within their respective categories for juvenile length gain (Supplementary Fig. 3) and were therefore compared against wheat gluten (category 2), defatted prepupae meal (category 4) and 9% flaxseed oil (FO) + 0.5% clove leaf oil (CLEO) (category 5) in the overall analysis^24,28,35,38^. The analysis demonstrated a significant effect of diet on juvenile length gain (one-way ANOVA: *F*_(4, 877)_ = 279.6, *P* < 0.0001) (Fig. 5c). Further analysis with Tukey’s multiple comparison test revealed an insect-based diet to be optimal for percentage length gain compared with the other feeding categories (Fig. 5c). The post-hoc comparison also revealed each diet to differ significantly between each other except for wheat gluten, which did not differ from 9% FO and 0.5% CLEO^24,28^.

## Discussion

In this systematic review, we originally aimed to determine the optimal feed for laboratory juvenile zebrafish growth and development by comparing growth parameters in published studies. Although we report that an insect-based diet resulted in the greatest increase in juvenile SGR, percentage weight gain and percentage length gain compared with the other tested categories, we caution the interpretation of this result owing to substantial caveats and limitations pertaining to the lack of sufficient reporting and variability in the experimental parameters of the included studies. Henceforth, the adapted aim of this Article was to provide an overview of the parameters that need to be standardized in future nutritional studies to facilitate future meta-analyses for confirmation of optimal juvenile diet for growth. Therefore, we believe the major finding from this present review is our reporting of the required standardized parameters for future nutritional juvenile zebrafish studies to facilitate confirmation of a standardized diet (Supplementary Table 2). Here, we describe our results and their caveats, as well as provide the required standardized parameters for future studies.

Our analysis incorporated three different growth parameters to ensure numerous aspects of juvenile growth were accounted for, including SGR, percentage weight gain and percentage length gain. SGR is a common growth parameter used in the zebrafish community that determines the growth (unit measurement nonspecific) per day^25^. This parameter was included to account for the large variability in the length of the feeding trial that we identified, which cannot be accounted for using weight and length gain (Fig. 2). Length and weight gain were also selected due to their commonality between the included studies. Although here we determined optimal growth to be the greatest increase in all three parameters, we do acknowledge that this may not necessarily constitute the optimal endpoint. Zebrafish width is an additional growth parameter that should have been assessed; however, there was a lack of reporting in the included studies. Width would have allowed a body condition scoring system to be implemented into the analysis, which has previously been used for laboratory zebrafish to assess their overall health and welfare^39^. The optimal width of zebrafish is reported to be around 810 mm (ref. ^39^). Comparatively, the optimal length of juvenile zebrafish ranges from 11.6 mm at 30 dpf to 19.5 mm at 89 dpf (ref. ^40^). Limited studies reporting optimal juvenile weight exist. Therefore, we encourage complete reporting of growth parameters (SGR, width, length and weight) for future nutritional studies using juvenile zebrafish (see Supplementary Table 2 for a full list of our recommendations).

Although our findings indicate a significant effect of including insect-based diets for juvenile zebrafish growth and development, we acknowledge substantial caveats to this finding. First, age can have a profound effect on growth responses^40,41^. Although steps were taken to reduce the likelihood of age influencing findings by providing a strict age range for the inclusion criteria, age still might have impacted the results. We chose to increase the age range included from 28 dpf to 4 months old, despite extending over the full age range of juvenile zebrafish (30–89 dpf)^42^. This choice was to overcome the limitations attributed to small study sizes when performing statistical analyses, which are known to cause erroneous results^42–45^. Therefore, the average start age between the included studies was 46 dpf, with the youngest age being 28 dpf and the oldest 120 dpf. (Table 1 and Fig. 2). A study performed by Singleman and Holtzman^41^ suggests that zebrafish growth is greater between the ages of 30 and 45 dpf compared with 45–60 dpf. However, the largest increase is seen between 90 and 180 dpf (ref. ^41^). Lanes et al.^35^ began their study at 30 dpf and therefore may have seen a greater increase in growth compared with the average starting age of 46 dpf, especially with a study length of 60 days and a final testing age of 90 dpf. However, the average length of the feeding trial and age at the end of the trial varied substantially between all analyzed studies (Fig. 2). The age at the end of the study ranged from 90 to 270 dpf, and the length of the trial ranged from 30 to 210 days. Clearly, this large variation in age will substantially impact the final growth performance and make interpretation of results challenging. Therefore, we encourage the start age of juvenile nutritional studies to be 30 dpf and final testing age to be 89 dpf. We also recommend regular growth parameter testing every week for accurate tracking of growth performance. These conditions ensure experimentation occurs only during juvenile stages and allows for effective comparisons to be made^14^.

Female zebrafish tend to grow heavier and be longer than male zebrafish^46,47^. Only half of the included studies reported the sex split within the feeding trial, and one of the studies that did report sex only used males^24^. This lack of sex reporting is commonly seen in the zebrafish research community, and this can have a considerable effect on inconsistencies between study findings^48^. Therefore, we strongly encourage future researchers to disclose the sex split of the fish and ensure an approximate 1:1 split throughout (survival dependent).

Although we do report survival in Supplementary Table 1, no statistical comparisons were made due to limited variability in survival regardless of diet. Studies have attempted to determine the effect of feed type and feeding frequency on survival, with little to no effect having been reported^25^. However, despite its limited effect on survival, feeding frequency can notably impact growth performance^15,25,37^. *The Zebrafish Book* reports feeding frequency to be two or more times in a day with enough feed for each fish to have food and it all to be gone within 5 min (ref. ^14^). Although a great reference point for zebrafish husbandry parameters, this lack of standardization and, hence, large variability in feeding frequency is apparent (Table 1). In addition, a common method for calculating feed amount is the use of the percentage of body weight. However, a recent systematic review reported that fish feeding according to body weight can have a substantially negative effect on growth performances^10^. Interestingly, 36% of the included studies used percentage of body weight during their feeding trials^10,25,27,28,30,31,36^. Therefore, this observation further highlights the importance of controlling these parameters when determining juvenile zebrafish growth and development. Thus, we conclude that, for future nutritional studies with juveniles, feeding should occur twice a day with enough food for each of the fish to eat and all to be eaten within 5 min (tank density dependent)^14^. However, we also encourage the reporting of the amount of food that ends up added to the tank per feed.

Additional identified factors that varied substantially between studies include fish strain, water quality and temperature, pH, population density and nutritional composition (see Supplementary Table 3 for nutritional composition)^41^. Although it is important that a variety of strains are included in nutritional studies for a biologically relevant finding, we do acknowledge that the use of different wild-type strains (unmodified, naturally occurring fish strains) can impact growth performance^49^. With this in mind, open reporting should take place regarding the strain of zebrafish used in future nutritional studies. However, no restrictions should be put in place on the strain used to ensure global experimental relevance^50^. Water temperature ranged from 24.87 to 28.5 °C in the included studies (Table 1). The optimal temperature for zebrafish is considered to be 28.5 °C, and therefore we suggest all future nutritional studies should be performed at 28.5 °C (ref. ^14^). pH also differed substantially between the studies; however, guidelines on zebrafish maintenance provide a wide range for pH, ranging from 6 to 8 (ref. ^51^). However, an optimal pH is considered to be 7.4; therefore, this value is what we would recommend for future nutritional studies^13^. In addition, the population density is optimal at approximately five fish per liter^52^. However, in the included studies, the tank density ranged from 1.2 to 18.0 per liter (Table 1). Tank density can affect growth factors, and therefore, all future nutritional studies should use five fish per liter with an approximate 1:1 sex split per tank. Four of the included studies did not report nutritional composition (Supplementary Table 3)^23,25,27,38^. Despite its importance, the lack of reporting by certain studies is concerning and highlights the importance of including all nutritional and ingredient information for future nutrition studies.

Therefore, although the significance of an insect-based diet as optimal diet for juvenile zebrafish growth and development cannot be confirmed due to the caveats mentioned, we do believe our findings are of critical importance to the zebrafish community because we highlight and provide the parameters required for future juvenile nutritional studies. We hope that the findings presented here encourage additional research using the standardized parameters, which will facilitate the determination of the optimal juvenile diet for growth as well as the dietary effects on longevity, fecundity, behavior and generational effects^10,53^.

## Conclusions

Our systematic review has brought to light the impact that a lack of experimental standardization can have on determining optimal juvenile nutrition and feeding for growth and development. The lack of standardized diet that we describe raises not only welfare considerations but also concerns relating to the reliability and reproducibility of research outcomes due to the substantial variability we found in growth responses with different diets. Therefore, we have highlighted the experimental parameters required to be standardized for future experimentation and conclude by encouraging future nutritional juvenile zebrafish research using the parameters described here.

## Methods

This systematic review was conducted in accordance with the Preferred Reporting Items for Systematic Reviews and Meta-Analyses (PRISMA) guidelines^54^. The checklists for both the article and abstract can be found in Supplementary Tables 4 and 5. All data and our analyses are accessible and downloadable on the Open Science Framework (https://osf.io/d5f7t/?view_only=8445745e4716467a9997bc62700ee67f).

### Search strategy

Searches were carried out in two bibliographic databases—PubMed and Scopus—using keywords that relate to our research topic for the intervention (fish feeds) and the desired population (laboratory zebrafish). Therefore, the following search string was applied: ‘Zebrafish AND (‘feed’ OR ‘diet’ OR ‘feeding’ OR ‘food’) AND (‘growth’ OR ‘survival’ OR ‘reproduction’ OR ‘welfare’). The search was carried out with no limitations on language or start date until August 2023. The reference lists of the included studies were also screened to detect additional relevant articles. The searches conducted and number of articles found are presented in Supplementary Table 6.

### Eligibility screening

After searching the databases, the selection of studies included in this review was performed by one independent researcher (C.H.) with regular discussions with a second reviewer (M.O.P.). Titles/abstracts were initially screened to identify and exclude duplicates. Thereafter, studies were selected on the basis of the inclusion and exclusion criteria (see below) by reading the titles/abstracts and, where necessary, the full text. Several studies had restricted access (paywalls), and the corresponding authors were contacted by email to request copies of the paper. They were given 14 days to provide the full text of the article, after which the study was excluded from the analysis.

Studies were included if they met the following criteria: (1) original experimental research performed with zebrafish ≥28 dpf testing different fish feeds or feeding regimens; (2) studies reporting growth (SGR, length and/or weight) and/or reproductive effects of fish feeds; (3) articles were peer-reviewed; (4) fish ≥28 dpf but 4 months old at the start of the feeding trial (growth analysis); (5) fish >4 months at the start of the feeding trial (reproductive analysis). Exclusion criteria were: (1) use of zebrafish <28 dpf or other research organisms; (2) studies that incorporated over- or underfeeding; (3) assessment of food supplements for translation into higher organisms; (4) review articles, retracted articles, book chapters, scientific letters and conference abstracts. We required a minimum of five studies for both growth and reproduction analyses. However, during this stage we discovered that there were too few reproduction-based studies that fit our inclusion criteria, and therefore, the reproductive analysis was removed (see ‘Search results’ section in Results for further information).

### Data extraction

One investigator (C.H.) worked independently to complete the data extraction, and consultation was provided, when necessary, by an additional reviewer (M.O.P.). All data extraction was performed from the full text and figures, and, where required, data were recalculated into the desired format (that is, percentage length gain was calculated from initial and final length data). The following information was collected: (1) general data: title, authors, publication year, age of fish, strain of fish, sex split in the experiment, sample size per experimental group (*n*), tank density, diet and feeding regimen before experimental diet, experimental diet and feeding regimen and husbandry conditions (Table 1); (2) growth results where applicable, including SGR, percentage weight gain, percentage length gain and percentage survival rate (Supplementary Table 1). Data were extracted as mean ± standard error of the mean, and when papers reported standard deviation, this was converted into standard error of the mean using an equation previously described in the literature and used in previous meta-analyses^55^.

Some studies only presented data in graphs (or not at all); therefore, authors were contacted via email to provide the additional data required for our analysis. These authors were given a 14-day period to respond; thereafter, PlotDigitizer (version 2.6.9. www.plotdigitizer.com) was used to manually estimate numbers from the graphs. Co-authorship networks were constructed using VOSviewer software version 1.6.20 (www.vosviewer.com)^33^.

### Risk of bias and reporting quality

To evaluate the quality of included studies, a risk-of-bias assessment was conducted by three independent investigators (C.H., A.H.C. and P.R.) for each paper. This analysis was performed using the Systematic Review Centre for Laboratory Animal Experimentation (SYRCLE) risk of bias tool for animal studies^56^. The risk of bias was assessed on the basis of the following: (1) random allocation of the animals; (2) comparable baseline groups; (3) allocation randomly concealed from researchers; (4) animals randomly housed; (5) confirmation of blinded investigators and/or caregivers; (6) animals selected at random for outcome determination; (7) description of investigator blinded during outcome assessment; (8) any incomplete data justified; (9) nonselective outcome reporting; (10) any other potential biases. The reporting was as follows: low (green), slight (light green), moderate (orange), high or unclear (red). Bias plots were created using GraphPad Prism 10 (GraphPad Prism version 10.1.2 for Windows, GraphPad Software, www.graphpad.com).

### Data analysis

The studies were split into three groups (SGR, percentage weight gain and percentage length gain) depending on data availability. Many of the identified studies were designed as a ‘control’ feed versus ‘experimental’ feed(s) experiment. Because there was no consistency between the ‘control’ diets, we further grouped the feeds together into six distinctive categories: (1) commercial feed; (2) plant-based protein; (3) animal-based protein; (4) insect-based protein; (5) supplements and additives; (6) combinations of feed/proteins. From this, where appropriate, a one-way ANOVA or unpaired Student’s *t*-test was performed to determine the most effective feed within the category using GraphPad Prism. For studies where more than one feed was available within a category, the feed reported as the most effective within the study was used for the analysis. A final one-way ANOVA was conducted combining the optimal feeds from each category to determine the overall optimal feed category for SGR, percentage weight gain and percentage length gain. Type 2 error rates were ****P < 0.0001; ***P < 0.001; **P < 0.01, *P < 0.05. Supplementary Fig. 4 provides a detailed breakdown of this analytical process.

## Online content

Any methods, additional references, Nature Portfolio reporting summaries, source data, extended data, supplementary information, acknowledgements, peer review information; details of author contributions and competing interests; and statements of data and code availability are available at 10.1038/s41684-024-01456-6.

## Supplementary information

Supplementary Information Supplementary Results, Figs. 1–4 and Tables 1–6.

## Data Availability

All data and analysis materials are available on the Open Science Framework (https://osf.io/d5f7t/?view_only=8445745e4716467a9997bc62700ee67f).
