# Supplementary material for: The effect of laboratory diet and feeding on growth parameters in juvenile zebrafish
Source: Lab Anim (NY). 2024 Oct 23;53(11):327–35. doi: 10.1038/s41684-024-01456-6 (PMC11518990; doi:10.1038/s41684-024-01456-6)
Supplement: Supplementary file 1 — Supplementary Results, Figs. 1–4 and Tables 1–6. [file 41684_2024_1456_MOESM1_ESM.pdf]

lab animal

Supplementary information

https://doi.org/10.1038/s41684-024-01456-6

The effect of laboratory diet and feeding on growth parameters in juvenile zebrafish

In the format provided by the authors and unedited

Supplementary Information

Supplementary Figures

Supplementary Fig. 1

The sub-group analysis findings for category 3 and 5 SGR are seen in Supplementary Fig. 1. Animal-based diets produced a significant effect on SGR between the studies (One-way ANOVA: $F_{(6, 360)} = 492.6$, $p < 0.0001$) (Supplementary Fig.1A). Lanes (2021) was found to produce the greatest increase in SGR compared to all other feeds with Tukey's multiple comparison test. No difference was seen between Sevgili (2018) and Fernandes (2016) with 50% FM. Similarly, no significant difference was seen between Vural (2021) and Dhanasiri (2020) with FM. Comparatively, the student's t-test determined no significant difference between 1.6% Royal Jelly compared to 6% FO and 0.5% CLEO in category 5 for SGR (student's t-test: $t(63) = 0.9485$, $p = 0.3465$) (Supplementary Fig. 1B) [26,36].

Supplementary Fig. 1 | The SGR sub-group analysis for categories 3 and 5.

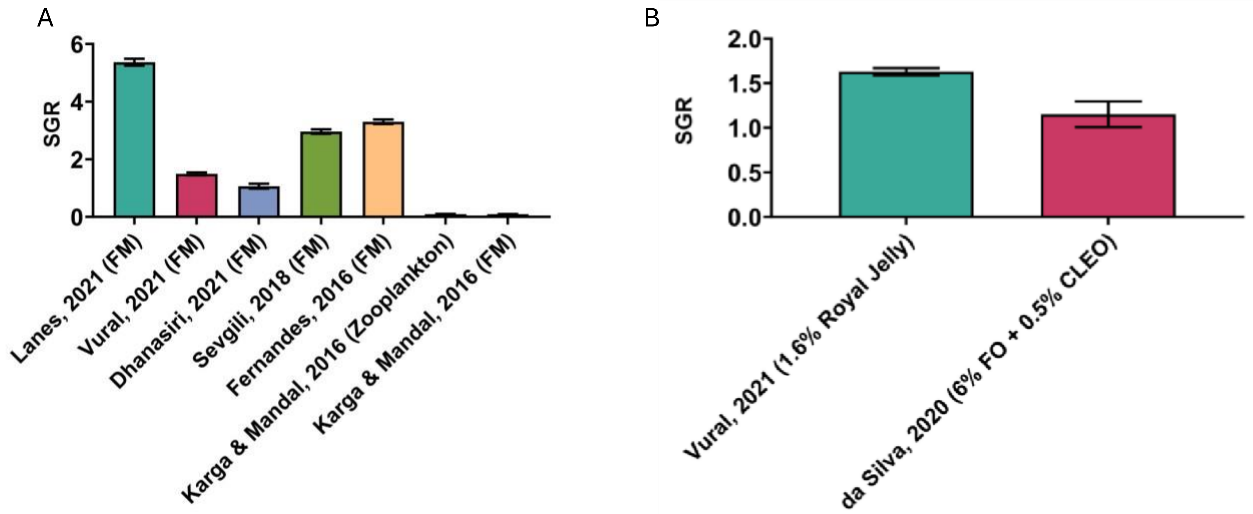

Note: The sub-group analyses for category 3 (A) and category 5 (B) SGR findings using a one-way ANOVA (A) and student's t-test (B). The significance values are not included on the graphs due to too many significance values. Please see the GraphPad file on OSF for full analysis results.

Supplementary Fig. 2

The sub-group analysis findings for categories 2-5 percentage weight gain are seen in Supplementary Fig. 2. A plant-based diet resulted in a significant effect on percentage weight gain with 100% *Chlorella sp.* causing a significant increase in

weight gain compared to soy-protein (student's t-test: t(83) = 15.44, $p < 0.0001$) (Supplementary Fig. 2A). An animal-based diet also caused a significant effect on overall percentage weight gain (one-way ANOVA: $F_{(9,\ 630)} = 24.28$, $p < 0.0001$) (Supplementary Fig. 2B). Tukey's multiple comparison test confirmed Lanes (2021) to produce the greatest increase in weight compared to all other studies. Lanes (2021) was also found to produce the greatest weight gain in category 4 (one-way ANOVA: $F_{(2,\ 237)} = 53.94$, $p < 0.0001$) (Supplementary Fig. 2C). No difference was seen between the two other insect-based diets. A significant effect on weight gain was seen in category 5 with 1.6% Royal Jelly significantly increasing percentage weight gain compared to a pro-biotic supplement (student's t-test: t(13) = 24.09, $p < 0.0001$) (Supplementary Fig. 2D).

Supplementary Fig. 2 |The sub-group analysis for categories 2-5 for weight gain (%).

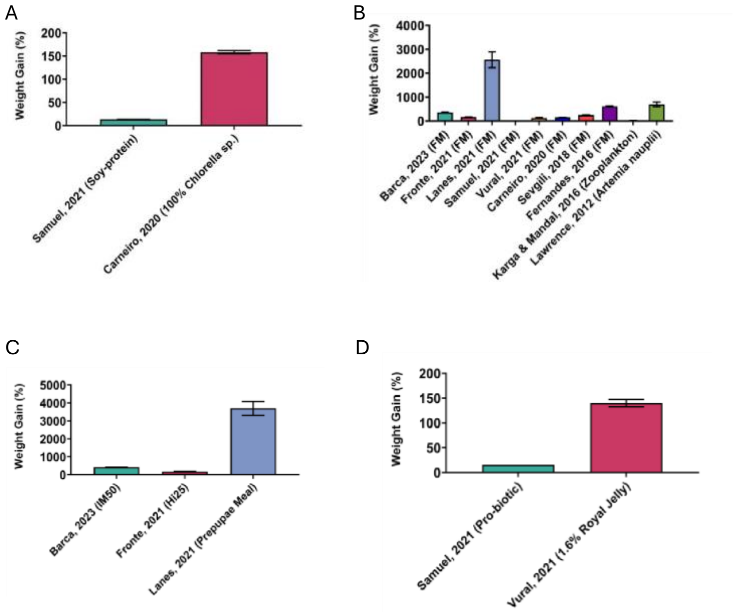

Note: The sub-group analyses for category 2 (A), 3 (B), 4 (C) and 5 (D) for weight gain (%) using a one-way ANOVA (B and C) and student's t-test (A and D). The significance values are not included on the graphs due to too many significance values. Please see the GraphPad file on OSF for full analysis results.

Supplementary Fig. 3

The sub-group analysis for category 3 and 6 for percentage length gain are seen in Supplementary Fig. 3. Lanes (2021) FM-based diet produced the greatest increase in percentage length gain (one-way ANOVA: $F_{(2, 167)} = 62.73$, $p < 0.0001$) (Supplementary Fig. 3A). The other FM-based diet also had significantly increased percentage length gain compared to the fish protein hydrolysate diet [28,30]. A mix of protein sources also significantly impacted length gain results (student's t-test: $t(620) = 10.83$, $p < 0.0001$) (Supplementary Fig. 3B).

Supplementary Fig. 3| The sub-group analysis for categories 3 and 6 for length gain (%).

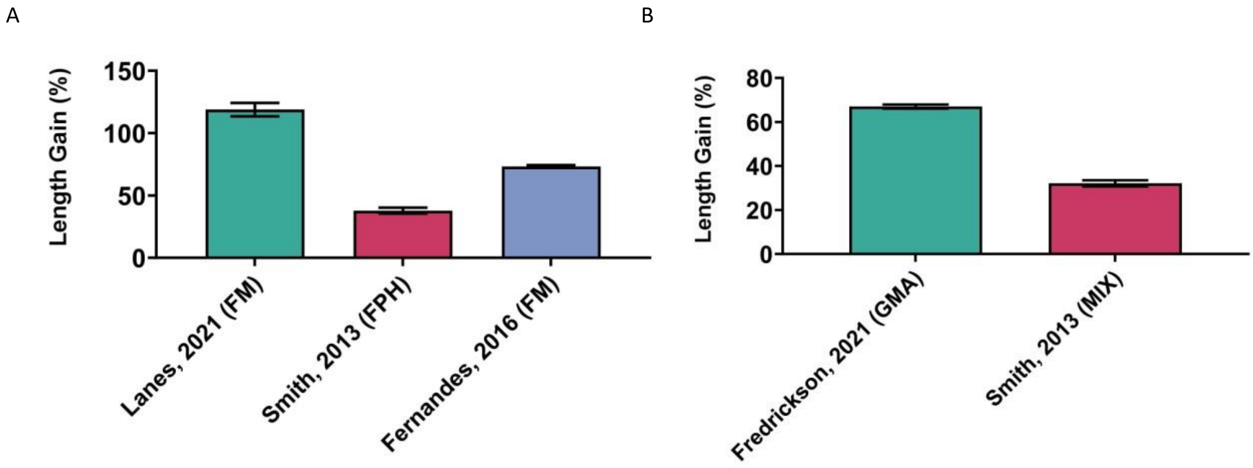

Note: The sub-group analyses for category 3 (A) and 6 (B) for length gain (%) using a one-way ANOVA (A) and student's t-test (B). The significance values are not included on the graphs due to too many significance values. Please see the GraphPad file on OSF for full analysis results.

Supplementary Fig. 4

The flow chart depicting the analytical steps for the statistical comparisons included in this analysis.

Supplementary Fig. 4 | Flow chart of analytical steps use for statistical comparisons.

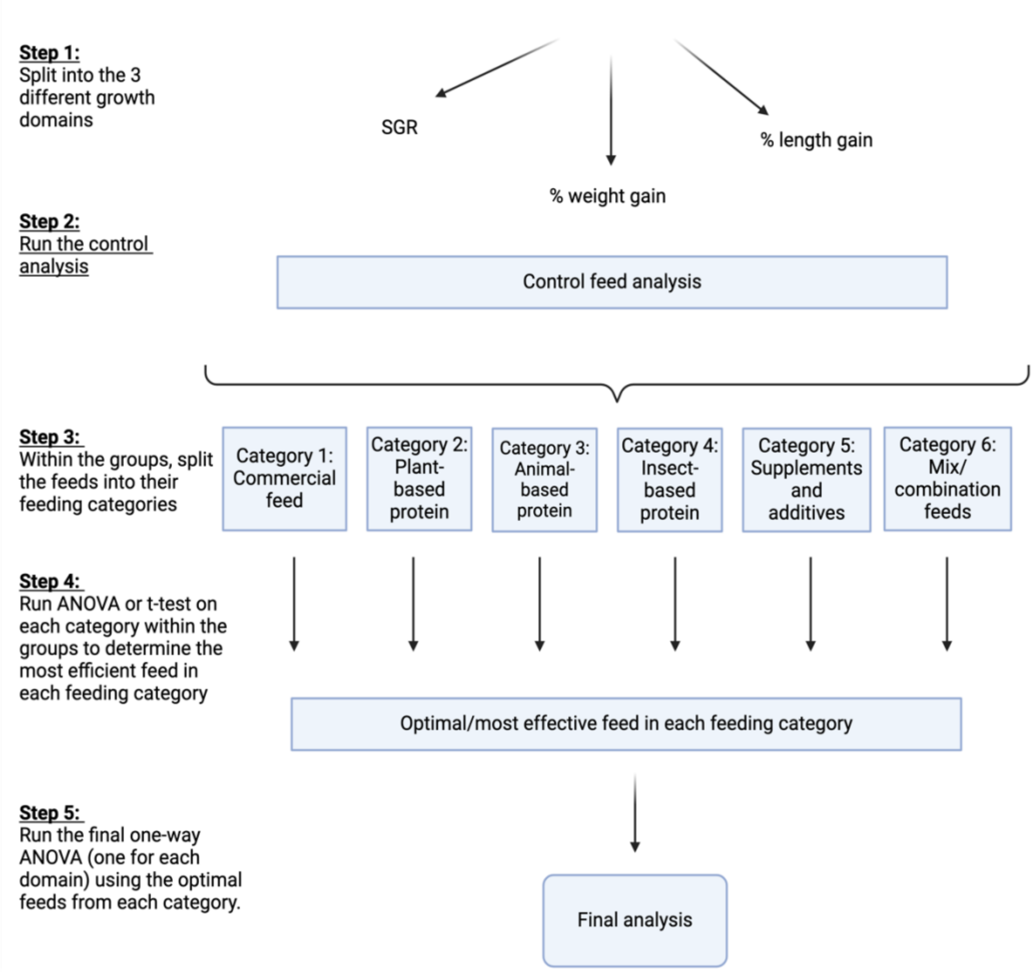

Note: An overview of the steps taken to perform the analysis presented in this paper. Figure was generated using Biorender.com.

Supplementary Tables

Supplementary Table 1

The statistical data that was used in the analysis included in this review is seen in Supplementary Table 1.

Supplementary Table 1 | The experimental data used in the current analysis from the studies describing the growth and survival effects of different feeds on juvenile zebrafish.

Author, year	SGR (% ± SE)	Weight gain (% ± SE)	Standard length gain (% ± SE)	Survival rate (% ± SE)
**Barca, 2023**		D1: 360.78 ± 7.68<br>D2: 392.25 ± 6.86<br>D3: 392.52 ± 7.09<br>D4: 417.91 ± 7.14		D1: 95<br>D2: 98.5<br>D3: 100<br>D4: 93.6
**Fronte, 2021**		D1: 169.64 ± 9.98<br>D2: 178.94 ± 10.44<br>D3: 181.63 ± 10.08<br>D4: 166.16 ± 9.74		D1: 88.75<br>D2: 92.5<br>D3: 88.75<br>D4: 93.75
**Lanes, 2021**	D1: 5.37 ± 0.12<br>D2: 5.67 ± 0.14<br>D3: 5.97 ± 0.04	D1: 2559.8 ± 20.81<br>D2: 3088.2 ± 26.45<br>D3: 3694 ± 10.07	D1: 118.91 ± 0.6986<br>D2: 137.39 ± 0.7431<br>D3: 150.87 ± 0.3970	D1: 92.50 ± 3.23<br>D2: 93.75 ± 4.73<br>D3: 96.25 ± 2.39
**Samuel, 2021**		D1: 14.38 ± 0.2<br>D2: 14.0 ± 0.01<br>D3: 15.7 ± 0.08<br>D4: 14.99 ± 0.11<br>D5: 13.27 ± 0.15		
**Vural, 2021**	D1: 1.49 ± 0.05<br>D2: 1.54 ± 0.09<br>D3: 1.07 ± 0.09<br>D4: 1.63 ± 0.04<br>D5: 1.54 ± 0.03	D1: 230.14 ± 6.90<br>D2: 237.04 ± 11.85<br>D3: 182.04 ± 8.80<br>D4: 249.11 ± 5.45<br>D5: 237.01 ± 4.12		D1: 79.33 ± 6.77<br>D2: 82.67 ± 5.81<br>D3: 88.0 ± 1.15<br>D4: 78.67 ± 10.48<br>D5: 84.00 ± 1.15
**Carneiro, 2020**	D1: 1.55 ± 0.0<br>D2: 1.61 ± 0.0<br>D3: 1.56 ± 0.0<br>D4: 1.55 ± 0.0<br>D5: 1.66 ± 0.0<br>D6: 1.58 ± 0.0	D1: 153.12 ± 2.3<br>D2: 163.87 ± 7.7<br>D3: 155.56 ± 2.5<br>D4: 154.27 ± 4.6<br>D5: 170.02 ± 2.9<br>D6: 158.39 ± 4.2		D1: 88.6 ± 3.6<br>D2: 94.3 ± 4.2<br>D3: 88.6 ± 2.9<br>D4: 88.6 ± 2.9<br>D5: 87.1 ± 3.5<br>D6: 91.5 ± 2.7

**da Silva, 2020**	D1: 0.46 ± 0.07<br>D2: 0.74 ± 0.15<br>D3: 0.78 ± 0.14<br>D4: 1.15 ± 0.15<br>D5: 0.85 ± 0.09<br>D6: 1.04 ± 0.13<br>D7: 0.93 ± 0.12		D1: 31.07 ± 1.65<br>D2: 33.23 ± 1.29<br>D3: 34.16 ± 1.59<br>D4: 34.79 ± 1.12<br>D5: 34.23 ± 1.29<br>D6: 36.38 ± 1.35<br>D7: 36.89 ± 1.02	D1: 100<br>D2: 100<br>D3: 100<br>D4: 100<br>D5: 100<br>D6: 100<br>D7: 100
**Dhanasari, 2020**	D1: 1.0679 ± 0.085<br>D2: 0.9119 ± 0.045<br>D3: 1.0157 ± 0.097<br>D4: 1.0756 ± 0.139			
**Sevgili, 2018**	D1: 2.90 ± 0.075<br>D2: 2.91 ± 0.075<br>D3: 3.05 ± 0.075<br>D4: 3.00 ± 0.075<br>D5: 3.02 ± 0.075<br>D6: 2.83 ± 0.075<br>D7: 2.96 ± 0.075<br>D8: 2.88 ± 0.075	D1: 238.22 ± 9.43<br>D2: 239.66 ± 9.47<br>D3: 260.00 ± 10.0<br>D4: 252.40 ± 10.10<br>D5: 254.81 ± 10.20<br>D6: 228.46 ± 10.17<br>D7: 247.05 ± 10.49<br>D8: 234.78 ± 10.35		
**Fernandes, 2016**	D1: 2.4 ± 0.08<br>D2: 2.6 ± 0.08<br>D3: 2.8 ± 0.08<br>D4: 2.9 ± 0.08<br>D5: 3.2 ± 0.08<br>D6: 3.2 ± 0.08<br>D7: 3.4 ± 0.08<br>D8: 3.3 ± 0.08<br>D9: 3.4 ± 0.08<br>D10: 3.3 ± 0.08	D1: 37.43 ± 2.85<br>D2: 40.00 ± 2.83<br>D3: 42.54 ± 2.93<br>D4: 43.55 ± 2.97<br>D5: 46.54 ± 2.88<br>D6: 47.34 ± 2.99<br>D7: 47.78 ± 3.00<br>D8: 46.79 ± 3.00<br>D9: 48.00 ± 3.03<br>D10: 47.29 ± 3.03	D1: 44.57 ± 0.705<br>D2: 54.35 ± 0.800<br>D3: 56.52 ± 0.826<br>D4: 58.70 ± 0.846<br>D5: 66.85 ± 0.920<br>D6: 66.30 ± 0.906<br>D7: 69.57 ± 0.937<br>D8: 73.37 ± 0.963<br>D9: 72.83 ± 0.958<br>D10: 69.02 ± 0.933	D1: 98 ± 1.04<br>D2: 93 ± 1.04<br>D3: 98 ± 1.04<br>D4: 100 ± 1.04<br>D5: 100 ± 1.04<br>D6: 100 ± 1.04<br>D7: 98 ± 1.04<br>D8: 93 ± 1.04<br>D9: 95 ± 1.04<br>D10: 95 ± 1.04
**Karga & Mandal, 2016**	D1: 0.097 ± 0.004<br>D2: 0.051 ± 0.005<br>D3: 0.089 ± 0.003<br>D4: 0.092 ± 0.004	D1: 22.62 ± 11.23<br>D2: 11.29 ± 4.82<br>D3: 20.41 ± 2.43<br>D4: 21.51 ± 4.17		D1: 86.67 ± 1.67<br>D2: 78.33 ± 3.33<br>D3: 83.33 ± 4.41<br>D4: 81.67 ± 1.67
**Smith, 2013**			D1: 5.85 ± 1.1097<br>D2: 5.44 ± 0.82<br>D3: 5.33 ± 1.1671<br>D4: 4.19 ± 0.5782<br>D5: 4.53 ± 0.8266	

**Lawrence, 2012**		D1: 723.09 ± 128.75<br>D2: 837.97 ± 32.67<br>D3: 900.69 ± 47.40<br>D4: 937.73 ± 79.38<br>D5: 221.46 ± 42.75	D1: 97.92 ± 27.18<br>D2: 110.17 ± 18.42<br>D3: 112.05 ± 9.89<br>D4: 109.11 ± 8.67<br>D5: 57.29 ± 13.77	D1: 96.9 ± 1.9<br>D2: 99.4 ± 0.6<br>D3: 98.8 ± 0.8<br>D4: 98.8 ± 1.6<br>D5: 98.8 ± 2.1

Supplementary Table 2

The suggested experimental parameters for future juvenile zebrafish nutritional studies determined from the analysis presented in this present review can be seen in Supplementary Table 2.

Supplementary Table 2 | The suggested experimental parameters for juvenile zebrafish nutritional studies.

Parameter	Our Recommendation
Age at the start of the study	30 dpf
Age at the end of the study	89 dpf
Feeding frequency	2x/day
Frequency of recording	1/week
Growth parameters to be recorded	SGR, width, weight, length, survival
pH	7.4
Population density	5/L
Reporting	Full reporting of all recordings and husbandry conditions to be provided in text or using an open-source framework
Sex split	1:1
Temperature	28.5°C

Note: Key; dpf: days post-fertilisation, L: litre, SGR: specific growth rate.

Supplementary Table 3

The nutritional composition of the different experimental feeds as reported in the included papers is seen in Supplementary Table 3.

Supplementary Table 3 | The nutritional composition (% inclusion) of the different feeds included in the present review.

Author, year	Dry matter (%)	Crude ash (%)	Crude lipid (%)	Crude protein (%)	Gross Energy (MJ/Kg)
**Barca, 2023***		D1: 8.07<br>D2: 7.09<br>D3: 6.15<br>D4: 5.13	D1: 13.61<br>D2: 14.77<br>D3: 15.73<br>D4: 16.75	D1: 58.84<br>D2: 58.36<br>D3: 58.53<br>D4: 58.42	D1: 20.91<br>D2: 20.91<br>D3: 20.77<br>D4: 20.62
**Frederickson, 2021**					
**Fronte, 2021**		D1: 5<br>D2: 4.7<br>D3: 4.4<br>D4: 3.8	D1: 15.2<br>D2: 15.5<br>D3: 15.9<br>D4: 15.5	D1: 60.4<br>D2: 60.0<br>D3: 60.2<br>D4: 59.8	D1: 21.9<br>D2: 21.9<br>D3: 21.8<br>D4: 21.6
**Lanes, 2021**	D1: 94.53<br>D2: 92.69<br>D3: 93.30	D1: 22.19<br>D2: 9.16<br>D3: 9.01	D1: 9.40<br>D2: 9.70<br>D3: 9.13	D1: 46.43<br>D2: 45.39<br>D3: 46.92	D1: 17.09<br>D2: 18.74<br>D3: 18.57
**Samuel, 2021**	D1: 90.0<br>D2: 92.0<br>D3: N/A<br>D4: N/A<br>D5: N/A	D1: 5.23<br>D2: 4.6<br>D3: N/A<br>D4: N/A<br>D5: N/A	D1: 4.1<br>D2: 3.9<br>D3: N/A<br>D4: N/A<br>D5: N/A	D1: 45.7<br>D2: 48.1<br>D3: 30<br>D4: N/A<br>D5:30	D1: 12.02<br>D2: 11.02<br>D3: N/A<br>D4: N/A<br>D5: N/A
**Vural, 2021**		D1: 3.84<br>D2: 3.84<br>D3: 3.84<br>D4: 3.84<br>D5: 3.84	D1: 14.25<br>D2: 14.25<br>D3: 14.27<br>D4: 14.34<br>D5: 14.63	D1: 45.68<br>D2: 45.69<br>D3: 45.74<br>D4: 45.92<br>D5: 46.65	
**Carneiro, 2020**	D1: 88.7<br>D2: 88.7<br>D3: 87.8<br>D4: 89.9<br>D5: 88.4<br>D6: 88.3	D1: 8.7<br>D2: 9.3<br>D3: 7.44<br>D4: 8.23<br>D5: 9.4<br>D6:10.0		D1: 32.3<br>D2: 32.7<br>D3: 31.9<br>D4: 32.5<br>D5: 32.3<br>D6: 31.6	D1: 16.7<br>D2: 16.7<br>D3: 16.7<br>D4: 16.7<br>D5: 16.7<br>D6: 16.7
**Da Silva, 2020**				D1: 51.82<br>D2: 51.26<br>D3: 51.15<br>D4: 50.97	D1: 17.16<br>D2: 17.28<br>D3: 17.57<br>D4: 17.23

				D5: 51.06<br>D6: 50.85<br>D7: 51.47	D5: 17.41<br>D6: 17.68<br>D7: 17.13
**Dhanasiri, 2020**			D1: 12.0<br>D2: 10.7<br>D3: 9.4<br>D4: 9.8	D1: 56.2<br>D2: 49.9<br>D3: 55.0<br>D4: 59.1	
**Sevgili, 2018**	20P: 91.41<br>25P: 91.74<br>30P: 91.64<br>35P: 91.89<br>40P: 92.31<br>45P: 92.24<br>50P: 92.66<br>55P: 92.49	20P: 5.75<br>25P: 6.73<br>30P: 6.94<br>35P: 7.63<br>40P: 8.21<br>45P: 8.98<br>50P: 9.71<br>55P: 10.51	20P: 10.26<br>25P: 10.02<br>30P: 10.32<br>35P: 10.94<br>40P: 10.62<br>45P: 9.97<br>50P: 10.28<br>55P: 10.34	20P: 20.38<br>25P: 26.22<br>30P: 28.93<br>35P: 34.94<br>40P: 39.56<br>45P: 44.2<br>50P: 49.72<br>55P: 56.88	20P: 19.82<br>25P: 19.69<br>30P: 19.7<br>35P: 19.81<br>40P: 19.74<br>45P: 19.47<br>50P: 19.58<br>55P: 19.62
**Fernandes, 2016**	15P: 89.2<br>20P: 90.1<br>25P: 91.3<br>30P: 92.4<br>35P: 90.6<br>40P: 93.1<br>45P: 94.3<br>50P: 93.8<br>55P: 94.2<br>60P: 93.6	15P: 10.9<br>20P: 11.2<br>25P: 12.1<br>30P: 12.9<br>35P: 13.2<br>40P: 14.2<br>45P: 14.6<br>50P: 15.4<br>55P: 15.8<br>60P: 16.5	15P: 8.7<br>20P: 8.9<br>25P: 8.4<br>30P: 8.0<br>35P: 8.8<br>40P: 8.7<br>45P: 8.8<br>50P: 9.4<br>55P: 10.0<br>60P:10.1	15P: 15.5<br>20P: 19.0<br>25P: 24.8<br>30P: 30.0<br>35P: 35.0<br>40P: 40.9<br>45P: 45.7<br>50P: 50.0<br>55P: 56.3<br>60P: 61.6	15P: 18.4<br>20P: 18.3<br>25P: 18.0<br>30P: 18.3<br>35P: 18.6<br>40P: 18.2<br>45P: 18.7<br>50P: 19.1<br>55P: 18.9<br>60P: 18.9
**Karga & Mandal, 2016**	D1: 9.62<br>D2: 90.52<br>D3: 90.36<br>D4: 90.1	D1: 6.15<br>D2: 3.41<br>D2: 3.29<br>D3: 3.11	D1: 12.95<br>D2: 3.86<br>D3: 4.58<br>D4: 5.41	D1: 54.18<br>D2: 35.12<br>D3: 39.76<br>D4: 44.40	D1: 17.34<br>D2: 14.02<br>D3: 15.66<br>D4: 15.71
**Smith, 2013**		D1: 6.84<br>D2: 8.37<br>D3: 7.89<br>D4: 6.86<br>D5: 5.95	D1: 12.34<br>D2: 15.86<br>D3: 10.47<br>D4: 10.33<br>D5: 11.10	D1: 44.9<br>D2: 45.3<br>D3: 44.5<br>D4: 43.9<br>D5: 49.5	

**Lawrence, 2012**					

Supplementary Tables 4 and 5

The PRISMA checklist for requirements of systematic reviews and meta-analysis is found in Supplementary Table 4 including the location in the present review and the abstract checklist is in Supplementary Table 5.

Supplementary Table 4 | The PRISMA checklist for systematic reviews and meta-analyses for this present review.

Section and Topic	Item #	Checklist item	Location where item is reported
**TITLE**			
Title	1	Identify the report as a systematic review.	1.0
**ABSTRACT**			
Abstract	2	See the PRISMA 2020 for Abstracts checklist.	Supplementary data
**INTRODUCTION**			
Rationale	3	Describe the rationale for the review in the context of existing knowledge.	1.0
Objectives	4	Provide an explicit statement of the objective(s) or question(s) the review addresses.	1.0
**METHODS**			
Eligibility criteria	5	Specify the inclusion and exclusion criteria for the review and how studies were grouped for the syntheses.	2.2

Information sources	6	Specify all databases, registers, websites, organisations, reference lists and other sources searched or consulted to identify studies. Specify the date when each source was last searched or consulted.	2.1
Search strategy	7	Present the full search strategies for all databases, registers and websites, including any filters and limits used.	2.1
Selection process	8	Specify the methods used to decide whether a study met the inclusion criteria of the review, including how many reviewers screened each record and each report retrieved, whether they worked independently, and if applicable, details of automation tools used in the process.	2.2
Data collection process	9	Specify the methods used to collect data from reports, including how many reviewers collected data from each report, whether they worked independently, any processes for obtaining or confirming data from study investigators, and if applicable, details of automation tools used in the process.	2.3
Data items	10a	List and define all outcomes for which data were sought. Specify whether all results that were compatible with each outcome domain in each study were sought (e.g. for all measures, time points, analyses), and if not, the methods used to decide which results to collect.	2.3
	10b	List and define all other variables for which data were sought (e.g. participant and intervention characteristics, funding sources). Describe any assumptions made about any missing or unclear information.	2.3
Study risk of bias assessment	11	Specify the methods used to assess risk of bias in the included studies, including details of the tool(s) used, how many reviewers assessed each study and whether they worked independently, and if applicable, details of automation tools used in the process.	2.4
Effect measures	12	Specify for each outcome the effect measure(s) (e.g. risk ratio, mean difference) used in the synthesis or presentation of results.	2.5
Synthesis methods	13a	Describe the processes used to decide which studies were eligible for each synthesis (e.g. tabulating the study intervention characteristics and comparing against the planned groups for each synthesis (item #5)).	2.5
	13b	Describe any methods required to prepare the data for presentation or synthesis, such as handling of missing summary statistics, or data conversions.	2.5
	13c	Describe any methods used to tabulate or visually display results of individual studies and syntheses.	2.5

	13d	Describe any methods used to synthesize results and provide a rationale for the choice(s). If meta-analysis was performed, describe the model(s), method(s) to identify the presence and extent of statistical heterogeneity, and software package(s) used.	2.5
	13e	Describe any methods used to explore possible causes of heterogeneity among study results (e.g. subgroup analysis, metaregression).	2.5
	13f	Describe any sensitivity analyses conducted to assess robustness of the synthesized results.	2.5
Reporting bias assessment	14	Describe any methods used to assess risk of bias due to missing results in a synthesis (arising from reporting biases).	2.5
Certainty assessment	15	Describe any methods used to assess certainty (or confidence) in the body of evidence for an outcome.	2.5
**RESULTS**			
Study selection	16a	Describe the results of the search and selection process, from the number of records identified in the search to the number of studies included in the review, ideally using a flow diagram.	3.1
	16b	Cite studies that might appear to meet the inclusion criteria, but which were excluded, and explain why they were excluded.	3.1
Study characteristics	17	Cite each included study and present its characteristics.	3.2
Risk of bias in studies	18	Present assessments of risk of bias for each included study.	3.3
Results of individual studies	19	For all outcomes, present, for each study: (a) summary statistics for each group (where appropriate) and (b) an effect estimate and its precision (e.g. confidence/credible interval), ideally using structured tables or plots.	Supplementary data

Results of syntheses	20a	For each synthesis, briefly summarise the characteristics and risk of bias among contributing studies.	3.4/5
	20b	Present results of all statistical syntheses conducted. If meta-analysis was done, present for each the summary estimate and its precision (e.g. confidence/credible interval) and measures of statistical heterogeneity. If comparing groups, describe the direction of the effect.	3.4/5
	20c	Present results of all investigations of possible causes of heterogeneity among study results.	5
	20d	Present results of all sensitivity analyses conducted to assess the robustness of the synthesized results.	N/A
Reporting biases	21	Present assessments of risk of bias due to missing results (arising from reporting biases) for each synthesis assessed.	3.3
Certainty of evidence	22	Present assessments of certainty (or confidence) in the body of evidence for each outcome assessed.	3.5/6
**DISCUSSION**			
Discussion	23a	Provide a general interpretation of the results in the context of other evidence.	4
	23b	Discuss any limitations of the evidence included in the review.	4
	23c	Discuss any limitations of the review processes used.	4
	23d	Discuss implications of the results for practice, policy, and future research.	4

**OTHER INFORMATION**			
Registration and protocol	24a	Provide registration information for the review, including register name and registration number, or state that the review was not registered.	Review was not registered
	24b	Indicate where the review protocol can be accessed, or state that a protocol was not prepared.	OSF
	24c	Describe and explain any amendments to information provided at registration or in the protocol.	N/A
Support	25	Describe sources of financial or non-financial support for the review, and the role of the funders or sponsors in the review.	declarations
Competing interests	26	Declare any competing interests of review authors.	The authors declare no competing interests
Availability of data, code	27	Report which of the following are publicly available and where they can be found: template data collection forms; data extracted from included studies; data used for all analyses; analytic code; any other materials used in the review.	OSF
and other materials			

Supplementary Table 5 | The PRISMA abstract checklist for systematic reviews and meta-analyses for the present review.

Section and Topic	Item #	Checklist item	Reported (Yes/No)
**TITLE**			
Title	1	Identify the report as a systematic review.	Yes
**BACKGROUND**			
Objectives	2	Provide an explicit statement of the main objective(s) or question(s) the review addresses.	Yes
**METHODS**			
Eligibility criteria	3	Specify the inclusion and exclusion criteria for the review.	Yes
Information sources	4	Specify the information sources (e.g. databases, registers) used to identify studies and the date when each was last searched.	Yes
Risk of bias	5	Specify the methods used to assess risk of bias in the included studies.	Yes
Synthesis of results	6	Specify the methods used to present and synthesise results.	Yes
**RESULTS**			
Included studies	7	Give the total number of included studies and participants and summarise relevant characteristics of studies.	Yes
Synthesis of results	8	Present results for main outcomes, preferably indicating the number of included studies and participants for each. If meta-analysis was done, report the summary estimate and confidence/credible interval. If comparing groups, indicate the direction of the effect (i.e. which group is favoured).	Yes

**DISCUSSION**			
Limitations of evidence	9	Provide a brief summary of the limitations of the evidence included in the review (e.g. study risk of bias, inconsistency and imprecision).	Yes
Interpretation	10	Provide a general interpretation of the results and important implications.	Yes
**OTHER**			
Funding	11	Specify the primary source of funding for the review.	(declarations)
Registration	12	Provide the register name and registration number.	N/A

773

Supplementary Table 6

The table including the searches conducted number of hits and number of articles found that are included in the analysis is reported on in Supplementary Table 6.

Supplementary Table 6 |The searches conducted, number of hits and the number of articles found which were included in the present analysis.

Date of Search	Search String	Database	Number of hits	Number of articles included in analysis
18/08/2023	Zebrafish AND diet AND growth	PubMed	285	6
21/08/2023	Zebrafish AND diet AND welfare	PubMed	15	0
21/08/2023	Zebrafish AND diet AND health	PubMed	212	0
21/08/2023	Zebrafish AND feed AND welfare	PubMed	14	0
21/08/2023	Zebrafish OR 'danio rerio' AND feed OR diet OR feeding AND welfare	Scopus	14	0
21/08/2023	zebrafish AND feed OR diet AND reproduction OR survival OR growth	Scopus	525	7
22/08/2023	N/A	citations and references	N/A	2
